# Stakeholder and Equity Data-Driven Implementation: a Mixed Methods Pilot Feasibility Study

**DOI:** 10.1007/s11121-022-01442-9

**Published:** 2022-10-04

**Authors:** Kelly A. Aschbrenner, Gina Kruse, Karen M. Emmons, Deepinder Singh, Marjanna E. Barber-Dubois, Angela M. Miller, Annette N. Thomas, Stephen J. Bartels

**Affiliations:** 1 Geisel School of Medicine at Dartmouth College, Hanover, NH USA; 2 Division of General Internal Medicine, Massachusetts General Hospital, Boston, MA USA; 3 Clinical Research Coordinator, Massachusetts General Hospital, Boston, MA USA; 4 Manet Community Health Center, Quincy, MA USA; 5 Lowell Community Health Center, Lowell, MA USA; 6 Brockton Neighborhood Health Center, Brockton, MA USA; 7 Department of Social & Behavioral Science, Harvard TH Chan School of Public Health, Boston, MA USA; 8 Massachusetts General Hospital, Boston, MA USA

**Keywords:** Health equity, Adaptations, Implementation, Mixed methods

## Abstract

We conducted a mixed methods pilot feasibility study of a Stakeholder and Equity Data-Driven Implementation (SEDDI) process to facilitate using healthcare data to identify patient groups experiencing gaps in the use of evidence-based interventions (EBIs) and rapidly adapt EBIs to achieve greater access and equitable outcomes. We evaluated the feasibility and acceptability of SEDDI in a pilot hybrid type 2 effectiveness-implementation trial of a paired colorectal cancer (CRC) and social needs screening intervention at four federally qualified community health centers (CHCs). An external facilitator partnered with CHC teams to support initial implementation, followed by the SEDDI phase focused on advancing health equity. Facilitation sessions were delivered over 8 months. Preliminary evaluation of SEDDI involved convergent mixed methods with quantitative survey and focus group data. CHCs used data to identify gaps in outreach and completion of CRC screening with respect to race/ethnicity, gender, age, and language. Adaptations to improve access and use of the intervention included cultural, linguistic, and health literacy tailoring. CHC teams reported that facilitation and systematic review of data were helpful in identifying and prioritizing gaps. None of the four CHCs completed rapid cycle testing of adaptations largely due to competing priorities during the COVID-19 response. SEDDI has the potential for advancing chronic disease prevention and management by providing a stakeholder and data-driven approach to identify and prioritize health equity targets and guide adaptations to improve health equity. ClinicalTrials.gov Identifier: NCT04585919.

Pursuing health equity in healthcare has been defined as striving to eliminate disparities in health and healthcare delivery between people who are more and less advantaged (Braveman, 2014). Implementation research that concentrates on factors, processes, and strategies for equitably implementing evidence-based interventions (EBIs) in routine care holds promise for closing healthcare gaps between those who are less and more advantaged based on social inequities (Baumann & Cabassa, 2020; Brownson et al., 2021). While implementation frameworks that have integrated a health equity lens provide a structure for evaluating equitable implementation (Shelton et al., 2020; Woodward et al., 2021), strategies are needed to identify health equity targets and rapidly design and test promising adaptations to address avoidable gaps in access and outcomes.

Stakeholder and data-driven implementation processes are promising approaches to advancing health equity. As health equity stakeholders, healthcare professionals can play an important role in selecting and defining patient groups to prioritize as health equity targets given their practice knowledge and understanding of local contexts. In addition, healthcare professionals can provide a critical perspective in designing EBI adaptations that are most likely to respond to local needs, experiences, and resources (Peters et al., 2017). Engaging healthcare professionals as stakeholders in designing and implementing processes that use data to identify and address gaps in healthcare access and health outcomes holds promise for advancing health equity (Chin, 2020).

Implementation facilitation is an evidence-based multi-faceted implementation strategy ideally suited to systematically guide processes for equitable implementation of EBIs (Harvey & Kitson, 2016). The facilitation process aims to transfer knowledge and skills from external facilitators to internal practice partners to build local capacity for implementation and sustainment efforts (Ritchie et al., 2020). Facilitators use quality improvement processes, including clinic workflow redesign and cyclical small tests of change, to address implementation challenges (Perry et al., 2019), which are promising strategies for rapidly designing and evaluating EBI adaptations.

## Purpose of the Present Study

We conducted a mixed methods pilot feasibility study of the SEDDI process where an external implementation facilitator collaborated with healthcare professionals to use data from a population health management system linked with electronic health records to identify patient subpopulations that experienced gaps in outreach and completion of a cancer screening EBI. Once gaps were prioritized as health equity targets, the external facilitator guided internal implementation teams to rapidly adapt, implement, and evaluate adaptations to address the inequities. We developed and evaluated SEDDI in a pilot hybrid type 2 effectiveness-implementation trial of a paired colorectal cancer (CRC) and social determinants of health (SDoH) screening intervention. This report presents mixed methods findings on the feasibility and acceptability of SEDDI. The clinical effectiveness outcomes for the paired screening intervention will be reported elsewhere.

## Methods

We conducted this study at the Harvard Implementation Science Center for Cancer Control Equity (ISCCCE), partnering with leadership and staff at four Federally Qualified Community Health Centers (CHCs) in Massachusetts. Harvard ISCCCE, in collaboration with the Massachusetts League of Community Health Centers (MLCHC), a Primary Care Association (PCA), has partnerships with a network of 30 CHCs across Massachusetts. Established under the same federal authorizing legislation as the health center program (Sect. 330 of the Public Health Service Act), PCAs are organized around a set of core functions and competencies that provide a framework for support and assistance to health centers and the communities they serve. MLCHC provides a wide range of technical assistance to CHCs, including workforce development, information technology development, and clinical quality initiatives. In this study, the MLCHC provided training and technical assistance to CHCs in obtaining and using data from a population health management system linked with electronic health records to identify and address inequities in cancer screening outreach and completion.

The paired CRC and SDoH screening intervention was implemented in a sequential, randomized rollout across the four participating CHCs over 8-week intervals. An external facilitator from Harvard ISCCCE partnered with local CHC implementation teams to support the initial 4-month implementation of the clinical intervention followed by a four-month SEDDI phase specifically focused on the equitable implementation of the intervention. In both phases, external facilitation was provided during 1-h bi-weekly or monthly virtual sessions over the 8-month study period. Preliminary evaluation of SEDDI involved convergent mixed methods with quantitative survey and focus group data integrated for comprehensiveness and to expand key findings. The Harvard Longwood Campus IRB approved study procedures with the Dana Farber IRB and Mass General Brigham IRB ceding review using the SMART IRB.

### Participants

The ISCCCE Implementation Laboratory (I-Lab) visited the four CHCs to introduce the clinical and implementation project to leadership and center staff (e.g., Chief Medical Officer and Population Health Manager) and asked each CHC to form an internal implementation team to participate in the paired screening implementation pilot. CHC participants (*N* = 21) on the implementation teams included quality improvement staff, community health workers, clinical staff (e.g., nursing, physicians, and medical assistants), population health and preventive services staff, and on-site laboratory services staff. These healthcare professionals were considered the “stakeholders” in this pilot feasibility study of SEDDI. The teams ranged in size from 4 to 7 members.

### Clinical Intervention

The clinical intervention consisted of paired CRC and SDOH screening targeting age-eligible (50–75 years old) average-risk adults who were not up to date on CRC screening with the goal of providing fecal immunochemical test (FIT) screening paired with screening for SDoH. Systematic reviews demonstrate the effectiveness of directly mailing a FIT to a patient’s home and patient reminders in increasing CRC screening rates (Dougherty et al., 2018; Issaka et al., 2019). Both CRC and SDoH screening activities were being delivered separately at the four CHCs and linked in the pilot hybrid type 2 effectiveness-implementation trial to address social needs exacerbated during the COVID-19 pandemic, which potentially impacted patients’ ability and willingness to engage in cancer screening. The clinical effectiveness outcome was CRC screening completion by any screening test (e.g., FIT, colonoscopy). The clinical effectiveness results will be reported elsewhere.

### Implementation Strategy

The integrated Promoting Action on Research Implementation in Health Services (i-PARIHS) framework was used to guide the implementation of the paired CRC and SDoH screening at CHCs. The i-PARIHS framework guides the assessment and alignment of a new innovation with the needs and preferences of recipients in their local, organizational, and wider system context (Harvey & Kitson, 2016). Implementation facilitators supported clinical teams in navigating change processes by addressing: (a) the new intervention’s fit within the existing clinic or practice; (b) the motivations, beliefs, goals, and resources of intervention recipients; (c) the inner and outer implementation context (Kirchner et al., 2014). A blended implementation strategy was used in which an external facilitator partnered with internal implementation teams formed for this study at each of the four CHCs to implement the paired screening intervention.

Prior to implementation, the external facilitator delivered a one-hour training to CHC teams that included education on the CRC and SDOH screening intervention and an overview of the planned implementation support. The overview covered: (1) outreach to eligible patients (could be linked to a visit or done by phone); (2) one-on-one education on the indications for, benefits of, and ways to overcome barriers to cancer screening; (3) education on completion of FIT and recommendations to offer other CRC screening tests based on patient preference and local clinical practices; and (4) SDoH screening. The CHCs were given an infographic that illustrated the paired screening intervention. MLCHC also provided training to CHCs about use of the population health management system to generate a registry of eligible patients for the paired screening intervention.

Following the training, quality improvement or population health staff from each of the four CHCs generated a registry of patients for outreach who were age-eligible adults (50–75 years old) due or overdue for CRC screening. They then narrowed the registry list in various ways tailored to the available resources and priorities of their respective CHCs, including patients who had completed FIT before but not in the past year; English-speaking only patients from racial groups with lower than average CRC screening rates; and patients scheduled for upcoming visits. Each CHC had an initial registry they used to make outreach calls to engage patients in the intervention.

The external facilitator met bi-weekly or monthly with CHC teams during the first four months of the project to review and support implementation progress. Core facilitation activities during the base implementation phase (first 4 months) included coaching and support; updates and feedback; guidance for workflow redesign; adapting the intervention to local context; and problem solving (Smith et al., 2020). During facilitations sessions, the external facilitator used a semi-structured guide developed by the research team to review progress; understand and identify barriers; help problem-solve and identify solutions; modify or adapt the implementation plan and prevent drift from the core elements of the EBI; and provide positive reinforcement, support, and encouragement to overcome barriers and challenges to implementation.

#### Stakeholder and Equity Data-Driven Implementation (SEDDI)

After 4 months of initial implementation support, the facilitation process transitioned to focus more specifically on advancing health equity through the SEDDI process. SEDDI was designed to help CHC implementation teams use their own data to identify patient subpopulations that experienced gaps in CRC screening outreach and completion. By identifying gaps, the clinical intervention could then be adapted or modified along with outreach strategies to address the inequities. Fifteen of the 21 CHC staff that participated in the base implementation phase participated in SEDDI, with some loss due to staff turnover. During SEDDI, the external facilitator continued to meet bi-weekly or monthly for one-hour virtual sessions with the CHC implementation teams.

**Fig. 1:**
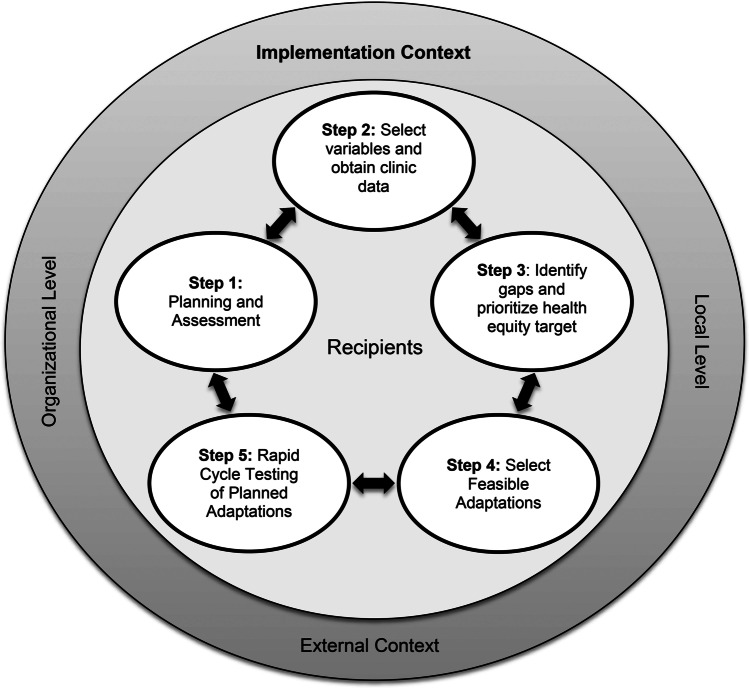
The SEDDI Process embedded in the integrated Promoting Action on Research Implementation in Health Services (i-PARIHS) framework

SEDDI (shown in Fig. 1) was modeled on elements of the dynamic adaptation process (DAP) (Aarons et al., 2012), a data-informed, collaborative, stakeholder-engaged approach to guiding adaptations to improve the fit of an EBI in a new context. The specific DAP elements applied to SEDDI included a pre-implementation assessment of system, organization, provider, and patient characteristics that were potential barriers and enablers to promoting equitable outreach, access, and use of the EBI; using results from the assessment to inform the selection of health equity targets; planning adaptations needed in the service context to address gaps and how such adaptations will be accomplished; and rapidly implementing and evaluating adaptations and making ongoing refinements as needed. The five steps in the SEDDI process are described below:

In step 1, the external facilitator provided CHC teams with an orientation to the SEDDI process and facilitated a discussion of potential barriers and enablers to promoting health equity in CRC screening and completion, including existing resources in the local setting that could be leveraged to address anticipated gaps in care.

In step 2, the external facilitator guided CHCs to download data from a population health management system linked to the electronic health record to make comparisons of (1) patient subgroups that were on the paired screening outreach list vs. the eligible population within these patient subgroups and (2) patient subgroups that returned the FIT kit vs. the outreach list to identify gaps in outreach and completion of CRC screening, respectively. The external facilitator guided the CHC implementation teams to select variables to define the patient subgroups for the comparisons, including patient race/ethnicity, language, and age and at least one additional demographic or characteristic, including gender identity and sexual orientation; income; insurance type; diagnosis; and zip code. The MLCHC provided technical assistance with the population health management system in the SEDDI phase by providing CHC implementation teams with a template and instructions to organize the data for the SEDDI comparisons.

In step 3, the external facilitator guided the CHC implementation teams to review the comparisons generated in step 2 to identify gaps in outreach and return of the FIT kit and prioritize health equity targets. The external facilitator examined the comparisons with the CHC teams, exploring their initial reaction, what stood out to them as a gap, and how large of a difference warranted consideration for adaptation. CHCs led the process of identifying meaningful differences in outreach and return of the FIT kit among patient subgroups based on their own context and settings. Gaps were prioritized as health equity targets warranting adaptation in outreach strategies or the paired screening intervention based on available resources and organizational priorities.

In step 4, the internal CHC implementation teams and the external facilitator discussed and identified adaptations to the outreach strategy and paired screening intervention to address identified gaps. The discussion included identifying existing resources (e.g., community health workers, interpretation, and translation services) that could be leveraged to support adaptations. The external facilitator guided the CHC implementation team to make adaptations that did not compromise the function of the paired screening intervention to promote CRC screening completion.

The initial plan for step 5 involved conducting a rapid cycle test of adaptation following a “Plan, Do, Study, Act (PDSA)” process. PDSAs test a change by planning it, trying it, observing the results, and acting on what is learned in short timeframes (Langley et al., 2009). The CHC implementation teams were to apply a PDSA by (1) planning a rapid-cycle test of the adaptation; (2) performing the test on a small scale; (3) analyzing the data and interpreting the results; and (4) refining the adaptation based on what was learned from the test and repeating the PDSA cycle, if necessary. The PDSAs were to be as brief as possible so additional modifications and refinements could be made, if necessary, to advance health equity.

### Pilot Study Evaluation

The pilot feasibility study of SEDDI involved convergent mixed methods with data from quantitative surveys and focus group interviews collected in parallel, analyzed separately, and then merged and integrated for completeness and to expand findings. Integrating for completeness allows a comprehensive understanding of a phenomenon by synthesizing quantitative and qualitative information, while expansion occurs when findings from the two sources of data describe divergent or complementary aspects of a central phenomenon (Bryman, 2006). In this mixed methods pilot feasibility study, the quantitative data addressed the feasibility and acceptability of SEDDI while the qualitative data added information about what, how, and why SEDDI was or was not feasible and/or acceptable and expanded findings beyond feasibility and acceptability of the initial model to include participants’ recommendations for refinements to the model.

#### Quantitative Data Collection

Implementation team members (*n* = 15) at each of the four CHCs that participated in SEDDI were asked to complete surveys for three brief quantitative measures of implementation outcomes: the acceptability of intervention measure (AIM), intervention appropriateness measure (IAM), and feasibility of intervention measure (FIM) (Weiner et al., 2017). AIM measured the degree to which SEDDI was satisfactory to CHC implementation teams. IAM measured the relevance or perceived fit of SEDDI. FIM assessed the degree to which SEDDI could be successfully used. Respondents were asked to indicate their level of agreement with statements on a 5-point Likert scale (1 = completely disagree, 5 = completely agree). Summary scores were created for each measure by averaging responses, with higher values reflecting more favorable perceptions of the SEDDI process.

#### Qualitative Data Collection

An experienced qualitative researcher led focus group interviews with each of the four CHC implementation teams following the SEDDI phase. The researcher moderated four 60-min focus group discussions using a topic guide that assessed: (1) CHC internal implementation teams’ experiences using SEDDI and receiving implementation support from the external facilitation team; (2) adaptations made to improve equitable outreach and use of the FIT kit; and (3) recommendations for refining the SEDDI process. A set of questions aligned with the Framework for Reporting Adaptations and Modifications-Expanded (FRAME) (Wiltsey Stirman et al., 2019) was used to elicit descriptions of the adaptations made to improve outreach and return of the FIT kit, including asking about the types of changes made, why the changes were made, who decided to make the changes, who delivered the changes, and how changes were delivered. The study coordinator took notes during the focus group interviews to enhance transcripts of the audio-recorded discussions.

The study coordinator also tracked adaptations to the paired screening intervention during facilitation sessions using a tracking log with a detailed description of the adaptation, including who made the adaptation, why the adaptation was made, and the date it was made. The research team further characterized the process and reasons for the adaptations applying the FRAME (Wiltsey Stirman et al., 2019).

### Data Analysis

Descriptive statistics (means and standard deviations) were computed at the item level and total scale level for each of the implementation outcome measures. The raw scores for each of the four CHCs were examined separately to explore potential heterogeneity and then combined for analysis.

A professional service transcribed focus group audio recordings. Data analysis involved an applied iterative coding process that uses both predetermined and emergent codes (Pope et al., 2000). The lead qualitative researcher developed an initial coding scheme based on topics assessed during focus groups, specifically: (1) CHC teams’ experiences using SEDDI and receiving implementation support from the external facilitation team; (2) adaptations made to improve equitable outreach and use of the FIT kit; and (3) recommendations for refining the SEDDI process. The coding scheme included concepts from FRAME (e.g., types of changes made, why the changes were made, and who decided to make the changes). Two researchers coded the data using the coding scheme while also looking for emergent codes. The researchers independently coded each of the four focus group transcripts and met after coding each transcript to review codes, discuss and resolve any disagreements about codes and code meaning, and agree on a final set of codes.

### Mixed Methods Data Integration

We merged the quantitative and qualitative data after the statistical analysis of the numerical data and qualitative analysis of the textual data (Fetters et al., 2013). Data integration involved a side-by-side comparison of the quantitative and qualitative findings using a summary table to add depth and comprehensiveness to the quantitative results (Creswell & Clark, 2017). We first integrated the data for completeness by synthesizing quantitative and qualitative data on feasibility, acceptability, and appropriateness of SEDDI and then integrated for expansion to capture, beyond initial feasibility, acceptability, and appropriateness, CHC teams’ recommendations for future research refinements to the SEDDI model (Bryman, 2006). Quotes that were representative of each of these two domains were selected for the report.

## Results

The number of facilitated SEDDI sessions at each of the CHCs ranged from 5 to 9 sessions, covering SEDDI steps 1–4 over 4 months. Each of the four CHCs obtained and used their own data to review gaps in the outreach list and return FIT kits for the paired screening intervention by race/ethnicity, gender, age, and language. Among the gaps identified, all four CHCs prioritized limited English proficiency as a health equity target. While none of the four CHCs advanced to step 5 (i.e., rapid-cycle testing of adaptations) during the defined project period due, in large part, to resource and time constraints caused by the COVID-19 pandemic, several CHC teams implemented and informally evaluated adaptations.

### Feasibility, Acceptability, and Appropriateness

As shown in Table 1, the SEDDI process was rated as highly feasible (*M* = 4.07, SD = 1.01), acceptable (*M* = 4.23, SD = 0.98), and appropriate (*M* = 4.23, SD = 0.68) by CHC internal implementation teams. The qualitative data provided insight into the high ratings of feasibility, acceptability, and appropriateness reported in the quantitative surveys. Specifically, CHC implementation teams shared how and why SEDDI was an appropriate and acceptable approach for identifying gaps and guiding adaptations. As one CHC implementation team commented:

“Having that structure and a way to do it was helpful for us, and then to be able to look at the data and bring it to our quality committee and take time to think about it, and then further investigate.”

“It was very helpful to have that data to then make the changes. We’re all in healthcare, so we’re all very much run by data. And so it was nice to have that structure and that template to go off of to help us out because I think we had a feeling. We knew certain departments weren't quite as successful as others. And it was nice to see the data back it up and help us make some changes.”

**Table 1:** Acceptability, appropriateness, and feasibility of the SEDDI process from the perspective of CHC implementation team members^a^

	**Mean (SD)**	**Proportion who agree or strongly agree**
**Acceptability**		
1. The guided adaptation support met my approval	4.33 (1.05)	93%
2. The guided adaptation support was appealing to me	4.20 (1.01)	93%
3. I liked the guided adaptation support	4.20 (1.01)	93%
4. I welcomed the guided adaptation support	4.20 (1.01)	93%
Total scale score	4.23 (0.98)	N/A
**Appropriateness**		
1. The guided adaptation support seemed fitting	4.20 (1.01)	93%
2. The guided adaptation support seemed suitable	4.13 (0.99)	93%
3. The guided adaptation support seemed applicable	4.33 (0.49)	100%
4. The guided adaptation support seemed like a good match	4.40 (0.51)	100%
Total scale score	4.23 (0.68)	N/A
**Feasibility**		
1. The guided adaptation support seemed implementable	4.13 (1.06)	87%
2. The guided adaptation support seemed possible	4.07 (1.03)	87%
3. The guided adaptation support seemed doable	4.07 (1.10)	80%
4. The guided adaptation support seemed easy to use	4.00 (1.00)	87%
Total scale score	4.07 (1.01)	N/A

^a^CHC implementation team members completed a brief web-based survey of 12, five-point Likert scale questions where 1 = strongly disagree, 2 = disagree, 3 = neither; agree nor disagree, 4 = agree, and 5 = strongly disagree

Another CHC team commented about the usefulness of the structure of SEDDI:

“Overall, the way that it was structured, especially from the standpoint of giving us the chance to look at the process post-adaptation—gave us the chance to iron out the workflow. Try something. If it doesn’t work, the process gave us the opportunity to make that adaptation and then fully implement. So I think it was great from the standpoint of having that extra eye asking, “What if?” or, “Why?”

Regarding feasibility, one CHC team commented that a transition in their electronic health record (EHR) was a barrier to accessing the data needed to examine gaps in care:

“The one stumbling block that we had with the data—at least in the latter half—was we changed EHRs, and so that made looking at the data a little bit more complicated because it wasn't integrated properly.”

One CHC team shared that addressing equity in a process like SEDDI often requires additional time and resources from providers and staff:

“Going to the population that we have, when that outreach, those phone calls are being made you’re not just making a phone call because the patient has 5 or 10 other things that, “Oh, while you’re on the phone, can you…” So that ends up taking a lot of time because maybe the patient has things that needed to be addressed but hadn't come in to be addressed.”

### Adaptations

As shown in Table 2, adaptations to the paired screening intervention included modifications to the content of the intervention, the way the intervention was delivered, and the way CHC staff were trained to deliver it. The primary reasons for the adaptations included cultural tailoring of a health message to recognize and reinforce a group’s cultural values, beliefs, and behaviors to provide context and meaning to a message (Resnicow et al., 2002); linguistic tailoring to make the intervention and materials more accessible by providing them in the dominant or native language of a target group (Kreuter et al., 2003); tailoring communication to the patient’s levels of health literacy (Schapira et al., 2017). One CHC implementation team described linguistic and health literacy tailoring of the FIT kit:

“One of the biggest gaps that we had identified was that the FIT Kit was not in any other language than English. It was not patient-friendly in the sense that when the patients were reading the instructions, the instructions weren’t clear enough. So that brought forth the collaboration between the lab and the medical assistant to change that workflow, change the card, translating it into different languages. And then we did see a drop in the number of FIT Kit that had errors.”

**Table 2:** Characterization of adaptations to the dual screening intervention

**Adaptation**	**Process**	**Reasons**
Delivered CRC screening education in-person or using telehealth (vs. phone outreach)	Modifications were made to the way the intervention was delivered	Tailored delivery to education and literacy levels and first/spoken languages
Modified pre-visit planning process	Modifications were made to the content of the intervention	Targeted patients due for FIT before an upcoming appointment
Used in-house interpreter during visits	Modifications were made to the way the intervention was delivered	Tailored delivery to first/spoken languages to improve language access
Modified outreach calls	Modifications were made to the way the intervention was delivered	Tailored delivery to cultural norms
Modified FIT materials	Modifications were made to the content/packaging of the intervention	Tailored materials for languages and literacy levels
Used language line services during outreach calls and CRC screening	Modifications were made to the way the intervention was delivered	Tailored delivery to first/spoken languages to improve language access
Added an extra follow-up phone call	Modifications were made to the way the intervention was delivered	Added an element to confirm patients received the FIT kit
Unpaired FIT and SDoH screening	Modifications were made to the way the intervention was delivered	Removed SDoH screening when it was redundant with services planned or received
Added provider-level intervention	Modifications were made to the way staff were trained to deliver the intervention	Added an element targeting certain providers to increase outreach to group with limited English proficiency

Another CHC team discussed their approach to targeting and training specific providers to improve outreach to patient groups experiencing gaps in care:

“I think we both kind of attribute it to one department that we know needs some assistance with the workflow. And they’ve continuously been a little bit behind. They have a different Chief. Whereas the other two departments that primarily do this have the same Chief. It’s been very successful. And so, I think we kind of suspected and it was like, “Oh, here’s the data to show it.” And we’re very fortunate that we do have a lot of Spanish-speaking staff. And so it’s definitely an area I think we can work on and see some progress with that with their help.”

### Recommended Refinements to SEDDI

The qualitative data expanded on the quantitative survey regarding the feasibility, acceptability, and appropriateness of SEDDI by eliciting CHC participants’ recommendations for future research refinements to the SEDDI process. CHC teams recommended that future research on SEDDI: (1) address equity early on in the implementation process; (2) include proximal outcomes of health equity; and (3) be selective in using rapid cycle tests during the COVID-19 pandemic due, in part, to workforce challenges and identify and train appropriate staff members to participate in the process.

One CHC implementation team suggested looking at data early in the implementation process rather than waiting until the SEDDI phase to prioritize equity:

“I think equity should be introduced earlier, so from the get go you’re really and truly looking into it…where it was at the beginning of the project, where it is in the middle of the project, and where we’re at in terms of the end of the project.”

Another CHC team discussed the importance of including proximal indicators of equitable implementation:

“If you’re talking to a patient that’s highly mistrustful of the medical system, then just at all getting them to engage is a huge success for that person. And I think that is very difficult to measure sometimes—we don’t track that because it's hard to quantify, but it is just as important, even more so sometimes, when you reach out to these particular patients and you just have a good conversation with them, and they have a little bit more of a positive experience with our health center.”

One CHC team cautioned that teams have needed to be selective in performing rapid cycle tests during the COVID-19 pandemic due, in part, to workforce challenges and emphasized the importance of identifying and training CHC staff members:

“Working in quality improvement, I have been able to do rapid cycle tests in the past, but you need to have the staff and the resources. The nurse practitioner would be willing to try something out for us, but beyond that, it would just be hard to get somebody on board to try different things and give us feedback…I think if we find the right staff member that we can engage and work with, definitely. And I think that would be very beneficial.”

## Discussion

This report presents results from a pilot feasibility study of the SEDDI process to help healthcare partners identify and prioritize health equity targets and rapidly design and test promising solutions to address gaps in healthcare delivery and outcomes. CHC teams rated SEDDI as highly acceptable, appropriate, and feasible. CHC implementation teams reported benefiting from the structured facilitation process targeting health equity, using data to identify and prioritize gaps in outreach and return of FIT kits. CHC teams adapted outreach strategies and the paired screening intervention to specifically address gaps among patients with limited English proficiency. Adaptations included cultural, linguistic, and health literacy tailoring of outreach messages and instruction materials for the FIT kit. None of the four CHCs advanced to step 5 (i.e., rapid-cycle testing of adaptations) during the defined project period due mostly to resource and time constraints caused by the COVID-19 pandemic. Recommendations to improve SEDDI included addressing health equity early on in the implementation process and using proximal outcomes of health equity. Recommendations for specific improvements to SEDDI included selectively using rapid cycle tests in consideration of staff burden and workforce challenges and identifying and training appropriate staff members to participate in the rapid cycle testing process.

Results from this pilot study demonstrated that SEDDI was an appropriate process for CHC settings. Federally Qualified Health Centers form the foundation of safety net primary care and, thus, health equity is central to the mission of CHCs. As our pilot study showed, using implementation facilitation to examine healthcare data can illuminate gaps in CRC screening among patient subgroups within this safety net. External facilitation has been used to train local staff to improve care processes in CHCs by using local practice knowledge to tailor an EBI to improve fit with a patient and provider’s needs and organizational capabilities (Fortney et al., 2018). Prior qualitative research with staff from 14 FQHCs in 8 states showed that few CHCs took a systematic approach to executing implementation plans for CRC screening and most did not actively target factors that influenced their CRC screening rates (Leeman et al., 2019). This research highlights opportunities for structured processes like SEDDI to identify patient groups experiencing inequities in outreach and use of EBIs and facilitate rapid adaptation of EBIs to achieve greater access and use.

CHC teams recommended that future research on SEDDI address equity early in the implementation process. Calls to advance health equity in implementation science have advocated for focusing on health equity at the earliest stages of implementation (Baumann & Cabassa, 2020; Brownson et al., 2021; Kerkhoff et al., 2022). The SEDDI pilot feasibility study was designed with input from CHC implementation teams to include a base implementation phase prior to starting the SEDDI process targeting health equity. CHC teams wanted to address initial implementation challenges such as integrating the pairing of CRC and SDoH screening into primary care workflows before making changes to the intervention to address inequities. However, upon reflecting on the process, CHC teams gave feedback that health equity should be addressed at the beginning of implementation to optimize the potential for reaching patients who are underserved by the CHC. This approach is consistent with recommendations to measure reach and effectiveness repeatedly to identify inequities throughout implementation (Shelton et al., 2020) and to use this information to guide early and mid-course adaptations to improve health equity (Glasgow et al., 2020).

Both the phased approach and the early approach to addressing health equity during implementation have merit. SEDDI is a process that can be used when teams are ready to adequately address and focus on targeting inequities, whether that is during the early phases of implementation or the sustainment phase. Addressing inequities at any stage of implementation will likely require additional training. For example, healthcare teams may require training on culturally tailoring outreach and/or interventions for certain groups to address inequities early on or later in the implementation process. Engaging healthcare stakeholders in study design and planning will help determine the optimal approach to addressing health equity and related training needs in a given context.

CHC teams emphasized the importance of identifying and training appropriate staff members to perform rapid cycle testing. We designed SEDDI to be similar to quality improvement processes that were familiar to CHC implementation teams, consistent with recent calls to design implementation strategies to fit into the existing culture, infrastructure, and practice of a healthcare system (Check et al., 2021; Leeman et al., 2021). Iterative, rapid cycle designs have a strong foundation in quality improvement science and are widely used to improve care quality and outcomes in healthcare settings (Lane-Fall & Fleisher, 2018). Healthcare stakeholders in this study cautioned the research team against assuming that CHCs have existing staff that is trained to perform rapid cycle testing of adaptations. While well-resourced learning health systems may have the capacity to use rapid cycle PDSA improvement methods to adapt EBIs (Chambers et al., 2016), using this approach may require initial training and ongoing technical assistance as part of an implementation strategy to successfully use rapid cycle testing in low-resourced healthcare settings pursuing health equity goals. With respect to the identification of proximal outcomes related to health equity evaluated during PDSA cycles, healthcare teams might consider consulting with a patient advisory board to identify patient-oriented proximal outcomes that are indicators of engagement from the patient’s point of view (e.g., patient satisfaction with outreach to diverse subgroups in implementing a new practice or intervention).

While stakeholder involvement beyond healthcare teams is strongly recommended as part of the SEDDI process, it is likely that selecting which stakeholders should be involved and their degree of involvement will be optimally determined by local implementation teams. For example, some healthcare teams may have access to patient advisory boards that could give input on specific adaptations needed to improve outreach efforts and/or the reach of interventions. In contrast, other healthcare teams may have access to providers and staff who themselves are family members or patients who experience healthcare inequities. These individuals could also represent these stakeholder perspectives in the SEDDI process. We have designed SEDDI with opportunities to incorporate stakeholder perspectives (e.g., providers, patients, and families) throughout each step of the process, while allowing flexibility for internal implementation teams to decide which stakeholder groups to involve based on perspectives needed to advance equity in the local context.

### Study Limitations

This pilot feasibility study was designed to assess the initial feasibility and acceptability of the SEDDI process. Aligned with the purpose of pilot research, this study focused on the SEDDI implementation process and addressed questions about whether and how the process could be implemented in routine care (Gadke et al., 2021). The clinical effectiveness outcomes for the paired screening clinical intervention will be reported elsewhere. Feasibility and acceptability data were collected from the perspective of CHC professionals who were stakeholders invested in SEDDI during the implementation of practice innovation. Future research evaluating SEDDI will investigate optimal approaches to engaging patients and community members as stakeholders in this process. There is a potential bias in this study toward implementation readiness among organizations that participated, given their ability to form internal implementation teams. Another limitation of this preliminary application of the SEDDI model was the CHCs’ inability to implement and evaluate rapid cycle testing of planned adaptations designed to address healthcare inequities.

### Conclusion

This mixed methods pilot feasibility study provides a basis for future research on implementation strategies for collaborating with healthcare partners to prioritize health equity targets and design and test promising adaptations to address gaps in healthcare delivery and outcomes. SEDDI was highly acceptable and feasible to implement from the perspective of CHC implementation teams. The model has the potential for advancing chronic disease prevention and management by providing a stakeholder and data-driven approach to identify and prioritize health equity targets and guide adaptations to improve implementation and health outcomes. Findings from this pilot study highlight important future research questions about the extent to which adaptations to EBIs can feasibly and effectively be rapid-cycle tested in low-resourced healthcare settings to promote equitable implementation. We look forward to our own research and the research of others applying and further refining the stages and process of the SEDDI model.
